# Low-Molecular-Weight Heparin Enhanced Therapeutic Effects of Human Adipose-Derived Stem Cell Administration in a Mouse Model of Lupus Nephritis

**DOI:** 10.3389/fimmu.2021.792739

**Published:** 2022-01-13

**Authors:** Shogo Matsuda, Takuya Kotani, Takashi Saito, Takayasu Suzuka, Tatsuhiko Mori, Tohru Takeuchi

**Affiliations:** ^1^ Department of Internal Medicine (IV), Osaka Medical and Pharmaceutical University, Takatsuki, Japan; ^2^ Department of Legal Medicine, Osaka Medical and Pharmaceutical University, Takatsuki, Japan; ^3^ Medical Education Center, Osaka Medical and Pharmaceutical University, Takatsuki, Japan

**Keywords:** adipose-derived stem cells, systemic lupus erythematosus, low-molecular-weight heparin (LMWH), lupus nephritis, anti-inflammation

## Abstract

**Background:**

Lupus nephritis is a life-threatening complication in systemic lupus erythematosus (SLE), but the efficiency of current therapies involving corticosteroids, immunosuppressants, and biological agents is limited. Adipose-derived mesenchymal stem cells (ASCs) are gaining attention as a novel treatment for inflammation in SLE. Low-molecular-weight heparin (LMWH) exhibits multiple functions including anti-inflammatory, anti-fibrotic, and cell function-promoting effects. LMWH stimulation is expected to increase the therapeutic effect of ASCs by promoting cellular functions. In this study, we investigated the effects of LMWH on ASC functions and the therapeutic effect of LMWH-activated human-ASCs (hep-hASCs) in an SLE mouse model.

**Methods:**

The cellular functions of human-derived ASCs stimulated with different LMWH concentrations were observed, and the optimum LMWH dose was selected. The mice were assigned to control, human-ASC, and hep-hASC groups; treatments were performed on week 20. Twenty-six week-old mice were sacrificed, and urine protein score, serum blood urea nitrogen, creatinine (Cr), anti-ds DNA IgG antibody, and serum IL-6 levels were analyzed in each group. Mice kidneys were evaluated *via* histological examination, immunohistochemical staining, and gene expression levels.

**Results:**

LMWH significantly promoted ASC migration and proliferation and hepatocyte growth factor production and upregulated immunomodulatory factors *in vitro*. Hep-hASC administration resulted in significant disease activity improvement including proteinuria, serum Cr and IL-6 levels, anti-ds DNA IgG antibody, glomerulonephritis, and immune complex in mice. Inflammation and fibrosis in kidneys was significantly suppressed in the hep-hASC group; the gene expression levels of TNF-alpha, TIMP-2, and MMP-2 was significantly downregulated in the hep-hASC group compared with the control group.

**Conclusions:**

Hep-hASC exhibited higher anti-inflammatory and anti-fibrotic effects than hASCs and may be a candidate tool for SLE treatment in future.

## Introduction

Systemic lupus erythematosus (SLE) is an autoimmune disease with multi-organ manifestations such as skin, lung, heart, and kidney (1). Lupus nephritis (LN) is one of the life-threatening complications in SLE, which correlates with high mortality rate (2). The efficiency of current therapies involving corticosteroids, immunosuppressants, and biological agents is limited for LN, and they have adverse side effects with long-term use (3). Therefore, new effective treatments for SLE are needed.

In animal models of LN and in clinical trials for refractory SLE, mesenchymal stem cells (MSCs) have been reported to be effective as a new therapy (4). Among MSCs, adipose tissue-derived mesenchymal stem cells (ASCs) exhibited a more potent inhibitory effect in preventing the activation of CD4+ and CD8+ T cells and NK cells than bone marrow-derived MSCs (BM-MSCs) and umbilical cord blood-derived MSCs (UC-MSCs) (5). In addition, ASCs have an advantage for therapeutic use because an adequate number of ASCs can be easily collected compared to BM-MSCS and UC-MSCs (6).

However, ASCs are more pro-coagulant than BM-MSCs, and ASCs cause a potential risk of pulmonary thromboembolism (PE) (7). BM-MSCs from SLE patients have reduced growth and differentiation activities *in vitro* compared with those from healthy individuals (8). If the cell functions of ASCs can be enhanced, it will be possible to 1) enhance the therapeutic effects of ASCs, 2) improve the impaired function of ASCs from SLE patients, and 3) reduce the number of ASCs used for treatment, leading to reduction in complications of PE.

Heparin binds to antithrombin (AT), accelerating the AT-mediated inhibition of coagulation factors (9). In addition to anticoagulant mechanism, heparin promotes production of hepatocyte growth factor (HGF) in various cells (10, 11). HGF binds to receptor tyrosine kinase c-MET and activates multiple downstream signaling pathways, such as the MAPK/ERK and PI3K/AKT pathways, leading to cell proliferation, cell growth, and survival (12). Furthermore, HGF has anti-inflammatory and anti-fibrotic effects in various organs (13, 14). Although bleeding acceleration is a side-effect of unfractionated heparin (15), low-molecular-weight heparin (LMWH) induces less bleeding (16). Moreover, the HGF secretion ability of LMWH is similar to that of unfractionated heparin (11, 17).

Therefore, we tested the hypothesis that LMWH-activated human-ASCs (hASCs) and HGF released from hASCs would exhibit a synergistically favorable effect on SLE by promoting anti-inflammatory and anti-fibrotic effects. In this study, the effects of LMWH on hASC functions were investigated, and the therapeutic effect of LMWH-activated hASCs was compared with that of hASCs alone in a mouse model of SLE.

## Materials and Methods

### Ethics

The Institutional Animal Care and Use Committee of Osaka Medical and Pharmaceutical University approved all of the following research protocols (approval ID: 2020-089), including the surgical procedures and animal care. All methods were performed in accordance with the relevant guidelines and regulations.

### Animal Models

Newborn female NZB ∕ WF1 mice were obtained after mating 8-week-old female NZB ∕ NSlc and male NZW ∕ NSlc mice (Shimizu Laboratory Supplies, Kyoto, Japan) in our laboratory.

### ASCs

Second-passage h-ASCs were purchased from Zen-Bio, Inc (Research Triangle Park, NC, USA) (No. BBASCF Lot. ASC012502). h-ASCs were cultured and used at the fifth-passage *in vivo* and the sixth-passage *in vitro*.

### Cell Migration Assay

The migration activity of hASCs was evaluated using a Boyden’s chamber assay. hASCs (50,000 cells per well) were seeded into the upper chambers of 24-well culture plates, and the lower chambers were filled with DMEM/F-12 medium containing 2% FBS and 1.0% Pen-Strep supplemented with 0, 1, 10, and 100 μg/mL of LMWH, followed by incubation for 6 h at 37°C. The migrated cells were stained with 4’,6-diamidino-2-phenylindole (DAPI) and counted in three randomly selected high-power fields (HPFs: ×200, 0.15 mm^2^ per HPF) per chamber under a fluorescence microscope, and the resulting numbers were averaged. The experiments were repeated five times independently for each sample.

### Cell Proliferation Assay

The proliferation activity of LMWH-activated ASCs was examined using a Cell Counting Kit-8 (Dojindo Laboratories, Kumamoto, Japan) according to the manufacturer’s instructions. Briefly, LMWH-activated ASCs were seeded onto 96-well culture plates at a density of 5,000 cells/100 µL per well. The cells were cultured in DMEM/F-12 containing 10% FBS and 1.0% Pen-Strep for 24 h at 37°C in a 5% CO2/95% air atmosphere. The medium was changed to the medium supplemented with LMWH (0, 1, 10, and 100 μg/mL) or without LMWH and cultured for 24 h at 37°C. The medium was subsequently exchanged with 100 µL of fresh medium containing 10% 2-(2-methoxy-4-nitrophenyl)-3-(4-nitrophenyl)-5-(2,4-disulfophenyl)-2H-tetrazolium (WST-8) of Cell Count Reagent SF and incubated for 30 min. The optical density at the 450 nm wavelength was measured using a plate reader. Cell proliferation activity was expressed as a ratio compared with cells cultured the media supplemented without LMWH. The experiment was repeated five times independently for each sample.

### HGF Immunoassay

Supernatants were evaluated for their HGF contents using an HGF Quantikine ELISA Kit (R&D Systems Inc., Minneapolis, MN, USA). The experiments were repeated four times independently for each sample and performed in duplicates.

### Quantitative Real-Time RT-PCR on hASCs *In Vitro*

hASCs were seeded onto 6-well culture plates (1 × 10^5^ cells per well), cultured for 24 h at 37°C in DMEM containing 1% FBS with or without LMWH. Total RNA was extracted from the cultured hASCs. Following RNA extraction with an RNeasy Mini Kit (Qiagen Ltd., Manchester, UK), cDNA was synthesized using an ExScript RT kit (Takara, Shiga, Japan), and amplification was performed on a ABI PRISM 7000 Sequence Detection System (Applied Biosystems, Tokyo, Japan) according to the manufacturer’s instructions. The primer sequences for CXC chemokine receptor 7 (CXCR7), CXC chemokine receptor 4 (CXCR4), HGF, CXCL12, phosphatidylinositol-3-kinase (PI3K), AKT, and glyceraldehyde 6-phosphate dehydrogenase (GAPDH) as a housekeeping gene are listed in **Supplementary Table 1**. Relative mRNA expression level of each target gene was calculated by the comparative C_T_ method, as described previously (18).

### Animals and Experimental Groups

LMWH-activated hASCs (hep-hASCs) were cultured in DMEM/F-12 containing 10% FBS and 1.0% Pen-Strep supplemented with LMWH (10 µg/mL). Twenty week-old female NZB/W F1 mice, showing proteinuria in urine test, were anesthetized with an intraperitoneal injection of 400 mg/kg 2,2,2-tribromoethanol (Avertin; Sigma-Aldrich Japan K.K., Tokyo, Japan). To assess the effects of LMWH-activated hASCs, the mice were randomly assigned to the following groups: (a) control (PBS-alone) group; (b) hASC-alone group; and (c) hep-hASC group (n=12 per each group). In the control group, 200 μL of PBS was injected intravenously. In the hASC group, hASCs were transplanted intravenously with 200 μL of PBS. In the hep-hASC group, hep-hASCs were transplanted intravenously with 200 μL of PBS. The number of transplanted cells was 1.0 × 10^4^ cells in the SLE mice. Following the treatments, the mice were euthanized at 26 weeks, when significant increase in mesangial cells and extensive mesangial matrix deposition was observed in glomerulus, and their kidneys were harvested for histological analysis (**Supplementary Figure 1**).

### Measurement of Proteinuria

During the experiment, urine protein levels were measured every 2 weeks. Fresh urine was collected by performing abdominal massage. The following system was used to score the severity of proteinuria, by testing urine on colorimetric Hema-Combistix strips (Siemens Healthcare Diagnostics K.K, Tokyo, Japan): 0 denotes trace value; 0.5 is 0–15 mg/dL; 1 is 30 mg/dL; 2 is 100 mg/dL; 3 is 300 mg/dL; 4 is 1000 mg/dL.

### Measurement of Blood Urea Nitrogen, Serum Creatinine, Anti-dsDNA Antibodies, and Serum Cytokine Level

Blood samples were collected from mice under isoflurane anesthesia at 26 weeks, and the sera were stored at −70°C until further assays. Serum blood urea nitrogen (BUN) levels were determined using a colorimetric assay (K024-H1; Arbor Assays, Ann Arbor, MI, USA). Serum creatinine in lupus mice was measured using the Creatinine Colorimetric/Fluorometric Assay kit (K625-100, BioVision, California, USA). Anti-ds DNA antibody levels were measured using a mouse anti-dsDNA ELISA kit (Shibayagi, Gunma, Japan). Serum IL-6 levels were measured using an ELISA kit (R&D Systems Inc., Minneapolis, MN, USA) (n=12 per each group). Serum BUN, Cr, anti-ds DNA antibody levels, and serum IL-6 levels were measured according to the manufacturer’s instructions.

### Histology

Kidneys were removed from 12 mice per group at 26 weeks. Paraffin-embedded kidney sections (2 µm) were stained with H&E/PAS/Masson. We performed histological examination of the glomeruli, tubulointerstitial, and vascular vessels, according to the previously established scoring system for kidneys of NZB/W F1 mice (19). In addition, we evaluated glomerular sclerosis using a previously described method (20). Histological examination was graded by two observers, and the scores were averaged.

### Immunofluorescence Staining for IgG, C3

Fresh kidneys were embedded in OCT compound and sectioned at 5 µm thick. They were fixed in ice-cold acetone for 5 min and washed thrice in phosphate buffer (0.05 M, pH 7.6). Nonspecific binding was blocked with Blocking One (03953-95; Nacalai Tesque, Inc.) for 30 min. The slides were drained and wiped with tissue paper. Next, the slides were incubated with FITC-conjugated goat anti-mouse IgG (1:100, AP 308F; Millipore) or FITC-conjugated goat anti-mouse C3 (1:100; CL7503F; Cedarlane) at room temperature for 1 h and then washed thrice in phosphate buffer (0.05 M, pH 7.6). After that, nuclei were counterstained with DAPI (Vector) and the slides were examined using a confocal laser scanning microscope BZ-X710 (Keyence). We evaluated a semiquantitative score of staining intensity distribution from 0 to 4 in a blinded manner as previously described (21).

### Quantitative Real-Time RT-PCR of Kidney *In Vivo*

Total RNA was extracted from the kidneys of mice at 26 weeks. The primer sequences for tissue inhibitor of metalloproteinase-1 (TIMP-2), matrix metalloproteinase-2 (MMP-2), TNF-α, IL-2, IL-4, IL-10, and GAPDH (as a housekeeping gene) are listed in **Supplementary Table 2**. The experiments were triplicated, and the results were averaged.

### Statistical Analysis

Statistical analyses were performed in GraphPad Prism version 7.0 software (GraphPad, San Diego, CA, USA) and JMP^®^ 14 (SAS Institute Inc., Cary, NC, USA). Statistical significance was determined using one-way analysis of variance with Dunnett’s multiple comparison test. Statistical significance was set at P<0.05.

## Results

### LMWH Enhanced the Cellular Functions of and HGF Production in hASCs *In Vitro*

To determine the optimum concentration of LMWM, the cellular functions of hASCs, such as the migration and proliferation abilities, were observed. In addition, HGF levels in the hASC culture supernatants were measured.

**Figure 1:**
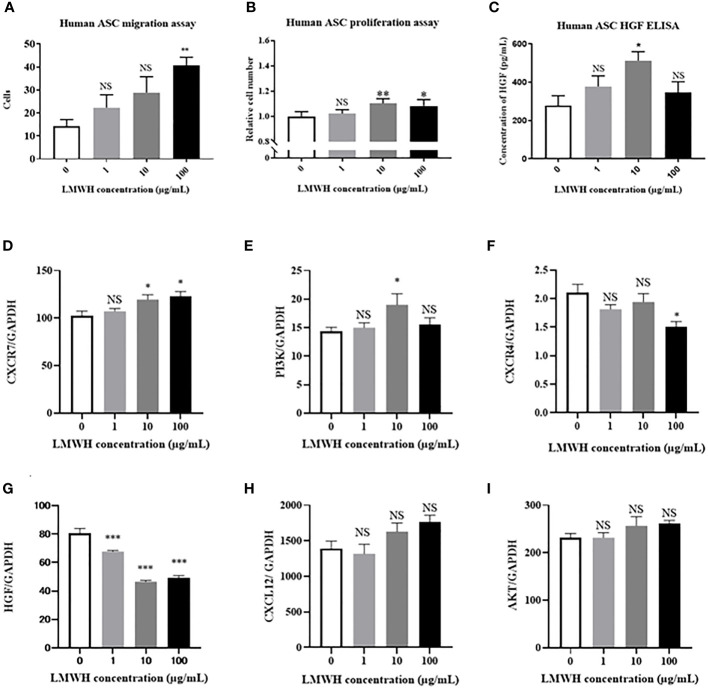
Effect of LMWH on human-ASCs. **(A)** The migration activity of human-ASCs (N=5 per group), **(B)** The proliferation activity of human-ASCs (N=5 per group), **(C)** The amount of HGF secreted by human-ASCs with or without LMWH were evaluated (N=4 per group). **(D–I)**. Immunomodulatory factors in hASCs with or without LMWH were evaluated as mRNA expression with quantitative real-time RT-PCR (N=6 per group). Data are shown as mean ± SEM. *P < 0.05. **P < 0.01. ***P < 0.001. NS, not significant vs. LMWH 0 group. ASCs, adipose-derived mesenchymal stem cells; CXCR, C-X-C motif chemokine receptors; HGF, hepatic growth factor.

We assessed the cellular functions of hASCs using different concentrations of heparin. The migration activity of hASCs was significantly higher in the 100 µg/mL LMWH concentration group than in the without LMWH concentration group (P=0.005) (**Figure 1A**). The proliferation activity of hASCs was significantly higher in the 10 and 100 µg/mL LMWH concentration groups than in the without LMWH concentration group (P=0.002 and 0.01, respectively) (**Figure 1B**). The amount of HGF secreted by hASCs was significantly higher in the 10 µg/mL LMWH concentration group than in the without LMWH concentration group (P=0.02) (**Figure 1C**).

### LMWH Regulated the Expression Levels of Genes Related to Immunomodulation

Immunomodulatory factors in LMWH-hASCs were evaluated in terms of mRNA expression using quantitative real-time RT-PCR (**Figures 1D–I**). In hASCs, the mRNA expression levels of CXCR7 were significantly upregulated in the 10 and 100 µg/mL LMWH concentration groups compared to the control group (*P*=0.03 and 0.01, respectively) (**Figure 1D**). In addition, the mRNA expression levels of PI3K were significantly upregulated in the 10 µg/mL LMWH concentration group compared to the control group (*P*=0.04) (**Figure 1E**). In contrast, compared to the control group, the mRNA expression levels of CXCR4 were significantly downregulated in the 100 µg/mL LMWH concentration group (*P*=0.006) (**Figure 1F**), and the mRNA expression levels of HGF were significantly downregulated in the 1, 10 and 100 µg/mL LMWH concentration groups (*P*=0.0007, <0.0001, <0.0001, respectively) (**Figure 1G**). The mRNA expression levels of CXCL12 tended to be upregulated in the 100 µg/mL LMWH concentration group compared to the control group (*P*=0.08); however, there were no significant differences (**Figure 1H**). From these results, 10 µg/mL of LMWH was determined to be the optimum concentration for maximizing the cellular functions of hASCs.

### Hep-hASC Administration Ameliorated Kidney Functions and Activity Indicators of SLE in NZB/W F1 Mice

**Figure 2:**
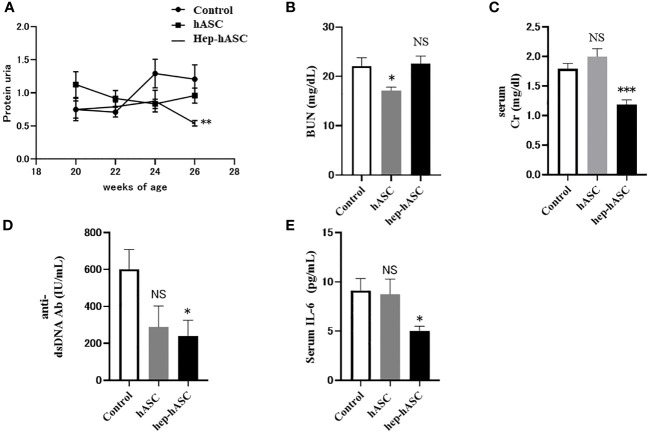
Effect of hep-hASC transplantation on clinical and serological parameters in NZB/W F1 mice. The effect of saline (control), hASC, and hep-hASC on **(A)** urine protein score, **(B)** serum blood urea nitrogen level, **(C)** serum creatinine level, **(D)** serum anti-dsDNA antibody titer, **(E)** serum IL-6 levels in NZB/W F1 mice. Data expressed as mean ± SEM (N=12 mice per group). *P < 0.05. **P < 0.01. ***P < 0.001. NS, not significant vs. control group. ASCs, adipose-derived mesenchymal stem cells.

Since LMWH significantly enhanced the cellular functions of hASCs, such as migration, proliferation, immunomodulatory gene expression, and anti-fibrosis effects, we investigated the effects of LMWH *in vivo* using an animal model of lupus. To investigate kidney functions and the levels of activity indicators of SLE, we measured urine protein score, serum BUN, Cr, anti-dsDNA antibodies, and IL-6. The urine protein score at 26 weeks was significantly lower in the hep-hASC group than in the control group (**Figure 2A**). There were no significant differences between the control and hASCs groups. Moreover, there were no significant differences in BUN levels between the hep-hASC and control groups (**Figure 2B**).The levels of serum Cr, anti-dsDNA antibodies, and IL-6 in the hep-hASC group were significantly lower (1.18 ± 0.08 mg/dL, 240.4 ± 85.1 IU/mL, and 5.0 ± 0.5 pg/mL, respectively) than those in the control group (1.79 ± 0.09 mg/dL, 601.6 ± 107.1 IU/mL, and 9.1 ± 1.2 pg/mL, respectively) (*P*=0.0005, 0.02, and 0.03, respectively) (**Figures 2C–E**). There were no significant differences in the levels of serum Cr, anti-dsDNA antibodies, and IL-6 between the control and hASCs groups.

### Hep-hASCs Transplantation Reduced Glomerulonephritis in the Kidneys of NZB/W F1 Mice

**Figure 3:**
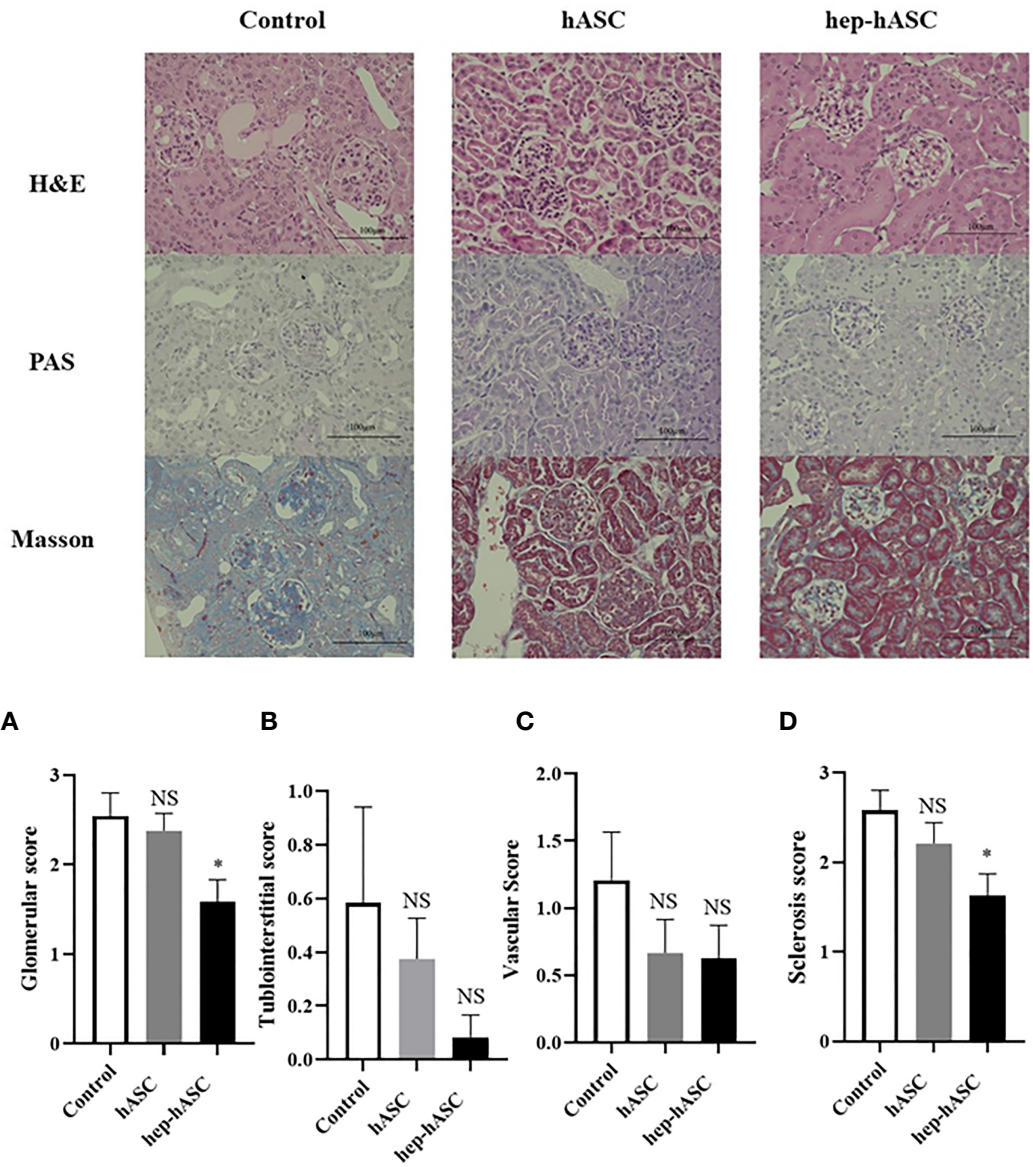
Effect of hep-hASC transplantation on renal histology in NZB/W F1 mice. Representative images showing renal histology in control, ASCs, and hep-ASCs transplanted mice at age 26 weeks (after disease onset) were shown. Hematoxylin and eosin (H&E), periodic acid–Schiff (PAS), Masson’s trichrome staining (×400) were evaluated. Renal histopathology included mesangial matrix deposition, crescent formation, urinary cast. Glomerular **(A)**, tubulointerstitial **(B)**, vascular **(C)**, and glomerulosclerosis score **(D)** were graded as described in *Material and Methods*. Data expressed as mean ± SEM (N=12 mice per group). *P < 0.05. NS, not significant vs. control group. ASCs, adipose-derived mesenchymal stem cells.

At 26 weeks, the kidneys isolated from the control group showed extensive mesangial matrix deposition and crescent formation. Periodic acid–Schiff (PAS) staining showed tubular casts indicating tubular injury. In addition, Masson trichrome staining showed increased matrix deposition indicating fibrosis. The glomerular, tubulointerstitial, vascular, and sclerosis scores in the kidneys were evaluated (n=12 per each group). The mean glomerular and sclerosis scores were significantly lower in the hep-hASC group than in the control group, although there was no significant difference between the control group and the hASC-alone group (**Figures 3A, D**). There were no significant differences in the tubulointerstitial and vascular scores between the control and hep-hASCs groups (**Figures 3B, C**). These results suggest that hep-hASCs prevent glomerulonephritis and glomerulosclerosis in NZB/W F1 mice.

### Hep-hASCs Transplantation Decreased Immunofluorescent Deposits in the Kidneys of NZB/W F1 Mice

**Figure 4:**
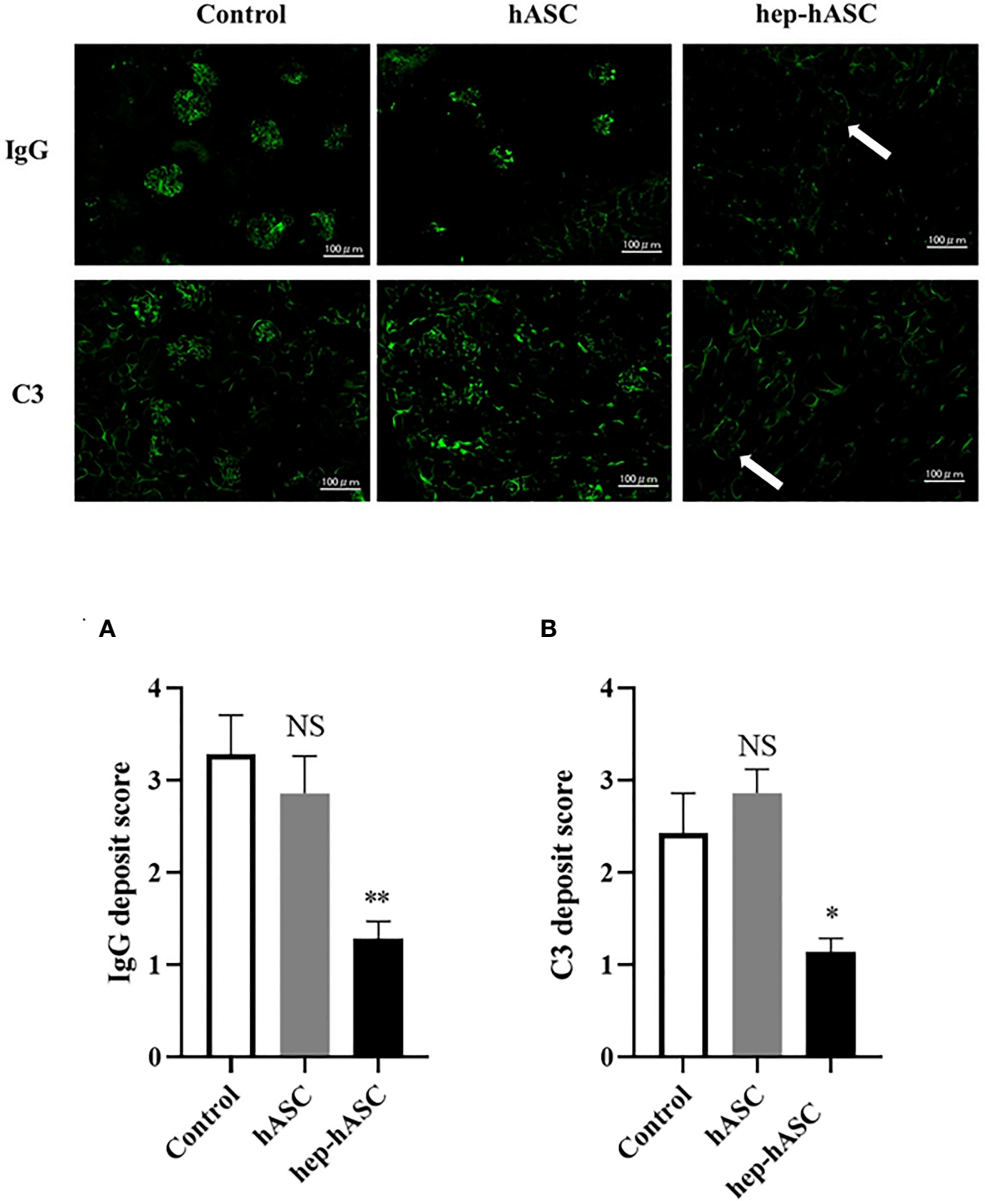
Effect of hep-hASC transplantation on IgG and C3 deposition in the kidneys of NZB/W F1 mice. Representative images showing renal IgG and C3 deposition in NZB/W F1 mice at age 26 weeks. Glomerular IgG **(A)** and C3 **(B)** deposition were graded as described in *Material and Methods*. Data expressed as mean ± SEM (N=7 mice per group). *P < 0.05. **P < 0.01. NS, not significant vs. control group. Original magnification ×200.

The fluorescence intensity of IgG or C3 deposits in the kidneys was evaluated (n=7 per each group). The mean fluorescence intensities of IgG and C3 deposits were significantly lower in the hep-hASC group than in the control group, although there were no significant differences between the control group and the hASC-alone group (**Figures 4A, B**).

### Hep-hASC Transplantation Regulated the Expression Levels of Genes Related to Tissue Inflammation and Fibrosis in the Kidneys of NZB/W F1 Mice

**Figure 5:**
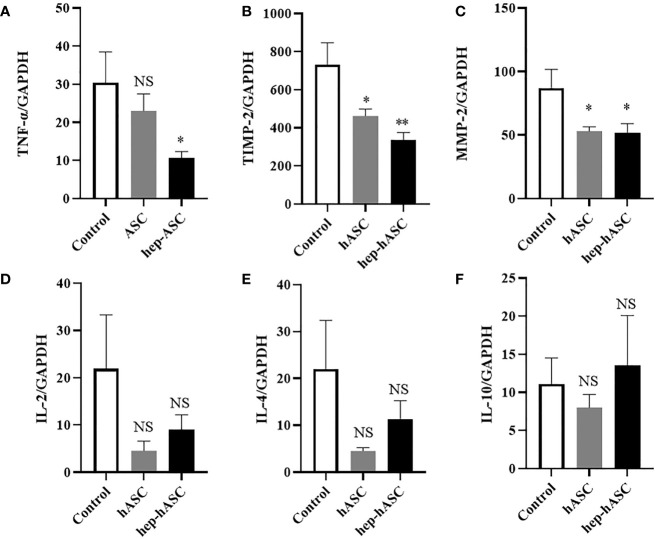
Quantitative real-time RT-PCR analysis of kidney mRNA expression. Relative mRNA expression levels of TNF-α **(A)**, TIMP-2 **(B)**, MMP-2 **(C)**, IL-2 **(D)**, IL-4 **(E)**, IL-10 **(F)** in the kidneys in NZB/W F1 mice at 26 weeks. Data are shown as mean ± SEM (N=12 mice per group). *P < 0.05. **P < 0.01. NS, not significant vs. control group.

To evaluate the anti-inflammatory and anti-fibrotic effects of hep-hASCs, mRNA expression levels of TNF-α, TIMP-2, and MMP-2 in the kidneys of NZB/W F1 mice were analyzed using quantitative real-time RT-PCR (n=12 per each group). The mRNA expression levels of TNF-α, TIMP-2, and MMP-2 in the hep-hASCs group were significantly lower than those in the control group (**Figures 5A–C**). In addition, there were no significant differences in mRNA expression levels of IL-2, IL-4, and IL-10 between the three groups (**Figures 5D–F**). These results suggest that hep-hASCs have anti-inflammatory and anti-fibrotic effects on nephritis in NZB/W F1 mice.

## Discussion

In our study, we demonstrated that higher concentration of LMWH (100 µg/mL) increases the migration abilities of hASCs. Additionally, higher concentrations of LMWH (10 and 100 µg/mL) increase the proliferation abilities of hASCs. High concentration of LMWH (10 µg/mL) also increases the amount of HGF secreted by hASCs. The mRNA expression levels of CXCR7 and PI3K were significantly increased in hASCs stimulated with high concentration of LMWH. Hep-hASC treatment group significantly reduced proteinuria, serum creatinine levels, anti-dsDNA antibody, and serum IL-6 levels compared to the control group in NZB/W F1 mice *in vivo*. In addition, C3 and IgG depositions in the glomerular spaces of the kidneys were significantly reduced in the hep-hASCs group than in the control group. The mRNA expression levels of TNF-a, TIMP2, and MMP2 were significantly lower in the hep-hASCs group than in the control group.

Ling et al. reported that BM-MSCs cocultured with heparin increased the proliferation and multipotentiality of hMSCs through the activation of FGF, BMP and Wnt signaling pathways (22). Also, Forte et al. reported that short-term exposure of mouse BM-MSCs to HGF can induce the activation of c-Met receptor and downstream effectors, such as ERK1/2, p38MAPK, and PI3K/Akt pathways, leading to the activation of BM-MSC migration (23).

In addition, The CXCL12/CXCR7 axis promotes MSC migration and proliferation activity (24), and CXCL12/CXCR7 signaling activates the ERK and PI3K/AKT pathways in human choriocarcinoma cells (25). However, the effect of heparin on hASCs has not yet been elucidated. In our study, the mRNA expression levels of CXCL12, CXCR7, and PI3K were upregulated in LMWH-activated hASCs. In addition, the mRNA expression levels of CXCL12 and CXCR7 in hASCs were positively correlated (**Supplementary Figure 2**). These findings suggest that the CXCL12/CXCR7 axis and PI3K/AKT pathways are related to the migration and proliferation activities in hep-hASCs. In contrast, the mRNA expression levels of CXCR4, a cell migration mediator, were significantly downregulated in hASCs stimulated with 100 µg/mL of LMWH. Ling et al. reported that high doses of heparin inhibited cell growth, and high doses of heparin may inhibit the cellular function of ASCs (22). Furthermore, in the present study, HGF secretion was upregulated in the hep-hASC groups stimulated with higher concentrations of heparin, but the mRNA expression levels of HGF were downregulated in hASCs stimulated with higher concentrations of heparin. Matsumoto et al. reported that LMWH increased HGF secretion without affecting the mRNA expression levels of HGF in the MRC-5 human fetal lung fibroblasts (10). Therefore, in our study, there may be a discrepancy between the protein level of HGF and the mRNA expression levels of HGF in hASCs.

The hep-hASC treatment group significantly improved glomerulonephritis compared to the hASC-alone group in NZB/W F1 mice *in vivo*. In the NZB/WF1 mouse model, IL-6 promotes the production of anti-ds DNA antibody from plasma cells, leading to immune complex glomerulonephritis (26). TNFα producing macrophages are involved in the pathology of glomerulonephritis (27). MSCs inhibit the differentiation of B cells into plasma cells, leading to the reduction of IL-6 in NZB/W F1 mice (28, 29). Moreover, MSCs inhibit the production of TNF-α produced by macrophages (30). In our study, the hep-hASC treatment group significantly reduced the levels of serum IL-6, serum ds-DNA antibody, and mRNA expression of TNF-α in mice kidneys compared to the hASC-alone group.

We also revealed that hep-hASCs significantly improved kidney fibrosis in NZB/WF1 mice. In the animal LN model, anti-inflammatory cytokines, such as TGF-β and IL-4, were overproduced to repair kidney inflammation, leading to fibrosis (31). Furthermore, serum TIMP-2 and MMP-2 levels were high in LN patients and mRNA expression levels of TIMP-2 and MMP-2 were upregulated in NZB/W F1 mice (32, 33). ASCs exhibit anti-fibrotic effects by downregulating the gene expression of pro-fibrotic markers and by upregulating the gene expression of anti-fibrotic factor (34). In our study, hep-hASCs significantly improved the fibrosis score in renal pathology and downregulated the mRNA expression of fibrotic synthesis factors in kidney.

HGF exhibits anti-inflammatory effects by disrupting the NF-kβ pathway, decreasing IL-6 production, and increasing IL-10 production from CD14+ monocytes through the ERK1/2 pathway in various cells (35–37). In addition, HGF exhibits anti-fibrotic effects by inhibiting fibroblast proliferation and TGF-β production from fibroblasts (38, 39). Therefore, in addition to the therapeutic effect of ASCs, HGF produced by hep-hASCs may have synergetic effects on LN by suppressing inflammation and fibrosis.

**Figure 6:**
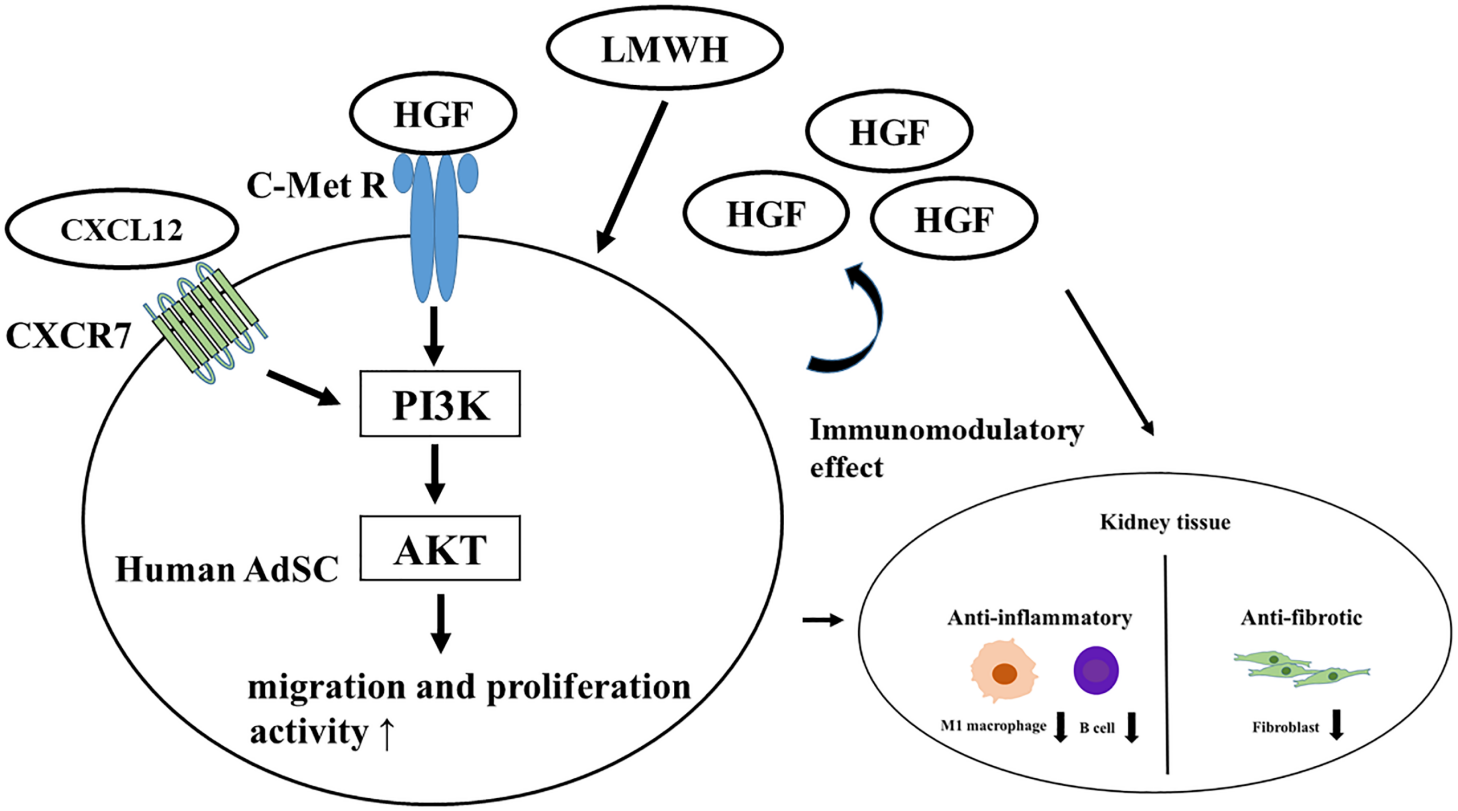
Therapeutic mechanism of hep-hASCs on lupus nephritis. LMWH promotes the production of HGF protein from hASC, and HGF activates PI3K/Akt pathway through c-MET receptor. Also, LMWH activates the CXCL12/CXCR7 pathway in hep-hASCs, leading to the activation of migration, proliferation, and homing capacity to kidney. In addition to activated cell function of hep-hASCs, increased amount of HGF protein from hep-hASCs has an anti-inflammatory and anti-fibrotic effect, leading to the improvement of LN. ASCs, adipose-derived mesenchymal stem cells; HGF, hepatic growth factor; PI3K, phosphatidylinositol-3-kinase; CXCR, C-X-C motif chemokine receptors.

The therapeutic mechanisms of hep-ASCs on SLE nephritis are presented in **Figure 6**. LMWH promotes the production of HGF protein from hASCs, and HGF activates the PI3K/Akt pathway through the c-MET receptor. In addition, HGF activates the CXCL12/CXCR7 pathway, leading to the activation of migration and proliferation activities of hASCs (40, 41). During these processes, hep-hASCs exhibit anti-inflammatory effects through the inhibition of B cells and macrophages and anti-fibrotic effects *via* inhibition of myofibroblasts in the kidney compared to the hASC-alone group. Besides the activation of cellular functions of hep-ASCs, increased HGF secretion by hep-ASCs strengthens the anti-inflammatory and anti-fibrotic effects, leading to the improvement of LN.

In preliminary experiments, we confirmed that the therapeutic effects on NZB/W F1 mice were obtained with 5×10^4^ hASCs, which is 5 times greater than the number of cells used in the present study (**Supplementary Figure 3**). LN can be treated with lower cell numbers of hASCs in our study because LMWH induce cellular activation of hASCs. Previous clinical studies reported that transplantation of 1×10^6^ MSC cells/kg exhibits remarkable therapeutic efficacy for LN (42–44). In our study, we used 1×10^4^ hep-hASCs in a 25 g mouse, which corresponds to 4×10^5^ cells/kg body weight. These cell numbers correspond to 40% of the cell numbers used in clinical practice. Although 1×10^4^ hASCs alone had no detectable therapeutic effect, same number of hep-hASCs demonstrated a remarkable therapeutic effect on the LN mouse model used in this study. This finding suggests that the LMWH-mediated increase in MSC function facilitates the reduction of MSC cell dose used for the treatment. Also, this provides an added advantage of minimal side effects, such as PE to this treatment strategy.

The renal histologies revealed that glomerulonephritis and glomerulosclerosis were ameliorated by hep-hASCs transplantation in NZB/W F1 mice, but no significant improvement was observed in cases of tubulointerstitial and vascular damage in our study. It has been previously reported that tubulointerstitial and vascular damage were less than glomerular damage in untreated NZB/W F1mice (45). Also, we observed mild tubulointerstitial and vascular damage in renal histologies of control groups, so there may not be any statistically significant difference in tubulointerstitial and vascular damage between the control and the hep-hASC groups.

There are several limitations in this study. First, the therapeutic effects of ASCs on LN may differ depending on the origin of ASCs; therefore, further studies are required to determine whether LMWH could enhance cellular functions of SLE-derived ASCs. Second, LN was improved by hep-hASCs administration in the early phase of our study. We elucidated the therapeutic effect of short-term hep-hASCs treatment, but the maximum duration till which the therapeutic effects of a single dose hep-hASCs can be retained remains elusive. Therefore, further investigations are warranted to estimate the long-term therapeutic effects of hep-hASCs treatment. Additionally, early-stage h-ASCs treatment was reported to exhibit superior therapeutic effects than an advanced-stage h-ASCs treatment (46); hence, further studies are required to determine whether LMWH-activated hASCs can improve LN in the progression phase. Third, it is imperative to consider the effect of reduction in time duration of the treatment by single-dosage of ASCs administration (47). To conclusively establish this effect, further research comparing the therapeutic effects of single hep-hASC administration to those of repeated hep-hASC administration on LN is required. Fourth, although we only evaluated the therapeutic effects of ASCs activated by 10 µg/mL LMWH, 100 µg/mL LMWH also increased migration and proliferation abilities of hASCs, and the mRNA expression levels of CXCR7 in hASCs. Therefore, further studies are required to evaluate the treatment efficacy of hASCs activated with 100 µg/mL LMWH. Finally, in this study, we could only evaluate the effects of hep-ASC treatment on the kidneys, hence in future, it would be interesting to study the therapeutic effects of hep-ASC on other organs as well.

## Conclusions

This study shows that hep-hASCs exhibit higher anti-inflammatory and anti-fibrotic effects than normal ASCs and may be a promising candidate tool for the treatment of LN in the future. The results of this study revealed a possible mechanism through which LMWH enhanced the therapeutic effects of hASCs, improved the impaired function of ASCs obtained from SLE patients, and reduced the number of hASCs used for treatment. Development of methods to further enhance the cellular functions of hASCs will be required.

## Data Availability Statement

The raw data supporting the conclusions of this article will be made available by the authors, without undue reservation.

## Ethics Statement

The animal study was reviewed and approved by The Institutional Animal Care and Use Committee of Osaka Medical and Pharmaceutical University (approval ID: 2020-089).

## Author Contributions

SM and TK contributed to the study design, data collection, and study execution, data analysis and interpretation, and preparation of the manuscript. TSa contributed to the study design (*in vitro* experiment), data collection, and study execution, data analysis, and preparation of the manuscript. TSu contributed to the data collection. TM contributed to the histological examination of kidney. TT helped to draft and approved the manuscript. All authors have read and approved the final manuscript.

## Conflict of Interest

The authors declare that the research was conducted in the absence of any commercial or financial relationships that could be construed as a potential conflict of interest.

## Publisher’s Note

All claims expressed in this article are solely those of the authors and do not necessarily represent those of their affiliated organizations, or those of the publisher, the editors and the reviewers. Any product that may be evaluated in this article, or claim that may be made by its manufacturer, is not guaranteed or endorsed by the publisher.

## Supplementary Material

Supplementary Figure 1 Experimental protocol. 20-week-old female NZB/W F1 mice were randomized to receive saline, hASCs alone and hep-hASCs and they were followed for a further 6 weeks. Mice were euthanized and blood, urine and kidneys were collected for clinical, serological, and histological analysis. ASCs, adipose-derived mesenchymal stem cells.

Supplementary Figure 2 Correlation of gene expressions in hASCs activated by LMWH. Correlation of CXCR7 mRNA expression levels with CXCL12 mRNA expression levels in hASCs. (N=24).

Supplementary Figure 3 Evaluation of therapeutic effects of ASC on lupus nephritis Comparison of kidney function between NZB/W F1 treated with 1×10^4^ hASC (N=12), 5×10^4^ hASC, (N=5) and 1×10^4^ hep-hASC (N=12). **(A)** Serum creatinine levels. **(B)** Serum anti-dsDNA antibody levels.

## References

[1] Rahman A Isenberg DA. Systemic Lupus Erythematosus. N Engl J Med (2008) 358:929–39. doi: 10.1056/NEJMra071297 18305268

[2] Maroz N Segal MS. Lupus Nephritis and End-Stage Kidney Disease. Am J Med Sci (2013) 346:319–23. doi: 10.1097/MAJ.0b013e31827f4ee3 23370533

[3] Fanouriakis A Kostopoulou M Alunno A Aringer M Bajema I Boletis JN. Update of the EULAR Recommendations for the Management of Systemic Lupus Erythematosus. Ann Rheum Dis (2019) 78:736–45. doi: 10.1136/annrheumdis-2019-215089 30926722

[4] Sattwika PD Mustafa R Paramaiswari A Herningtyas EH. Stem Cells for Lupus Nephritis: A Concise Review of Current Knowledge. Lupus (2018) 27:1881–97. doi: 10.1177/0961203318793206 30099942

[5] Ribeiro A Laranjeira P Mendes S Velada I Leite C Andrade P. Mesenchymal Stem Cells From Umbilical Cord Matrix, Adipose Tissue and Bone Marrow Exhibit Different Capability to Suppress Peripheral Blood B, Natural Killer and T Cells. Stem Cell Res Ther (2013) 4:125. doi: 10.1186/scrt336 24406104 PMC3854702

[6] Planat-Benard V Silvestre JS Cousin B André M Nibbelink M Tamarat R. Plasticity of Human Adipose Lineage Cells Toward Endothelial Cells: Physiological and Therapeutic Perspectives. Circulation (2004) 109:656–63. doi: 10.1161/01.CIR.0000114522.38265.61 14734516

[7] Toyserkani NM Jørgensen MG Tabatabaeifar S Jensen CH Sheikh SP Sørensen JA. Concise Review: A Safety Assessment of Adipose-Derived Cell Therapy in Clinical Trials: A Systematic Review of Reported Adverse Events. Stem Cells Transl Med (2017) 6:1786–94. doi: 10.1002/sctm.17-0031 PMC5689766 28722289

[8] Sun LY Zhang HY Feng XB Hou YY Lu LW Fan LM. Abnormality of Bone Marrow-Derived Mesenchymal Stem Cells in Patients With Systemic Lupus Erythematosus. Lupus (2007) 16:121–8. doi: 10.1177/0961203306075793 17402368

[9] Casu B Naggi A Torri G. Re-Visiting the Structure of Heparin. Carbohydr Res (2015) 403:60–8. doi: 10.1016/j.carres.2014.06.023 25088334

[10] Matsumoto K Tajima H Okazaki H Nakamura T. Heparin as an Inducer of Hepatocyte Growth Factor. J Biochem (1993) 114:820–6. doi: 10.1093/oxfordjournals.jbchem.a124262 8138538

[11] Saito T Kotani T Suzuki K. Antifibrotic Therapy by Sustained Release of Low Molecular Weight Heparin From Poly(Lactic-Co-Glycolic Acid) Microparticles on Bleomycin-Induced Pulmonary Fibrosis in Mice. Sci Rep (2020) 10:19019. doi: 10.1038/s41598-020-76034-0 33149192 PMC7642430

[12] Noriega-Guerra H Freitas VM. Extracellular Matrix Influencing HGF/c-MET Signaling Pathway: Impact on Cancer Progression. Int J Mol Sci (2018) 19:3300. doi: 10.3390/ijms19113300 PMC6274944 30352967

[13] Gong R Rifai A Dworkin LD. Anti-Inflammatory Effect of Hepatocyte Growth Factor in Chronic Kidney Disease: Targeting the Inflamed Vascular Endothelium. J Am Soc Nephrol (2006) 17:2464–73. doi: 10.1681/ASN.2006020185 16885407

[14] Schievenbusch S Strack I Scheffler M Wennhold K Maurer J Nischt R. Profiling of Anti-Fibrotic Signaling by Hepatocyte Growth Factor in Renal Fibroblasts. Biochem Biophys Res Commun (2009) 385:55–61. doi: 10.1016/j.bbrc.2009.05.010 19426716

[15] Turpie AG Gallus AS Hoek JA Investigators P. A Synthetic Pentasaccharide for the Prevention of Deep-Vein Thrombosis After Total Hip Replacement. N Engl J Med (2001) 344:619–25. doi: 10.1056/NEJM200103013440901 11228275

[16] Schulman S Beyth RJ Kearon C Levine MN. Hemorrhagic Complications of Anticoagulant and Thrombolytic Treatment: American College of Chest Physicians Evidence-Based Clinical Practice Guidelines (8th Edition). Chest (2008) 133(6 Suppl):257S–98S. doi: 10.1378/chest.08-0674 18574268

[17] Sakiyama R Fukuta K Matsumoto K Furukawa M Takahashi Y Nakamura T. Stimulation of Hepatocyte Growth Factor Production by Heparin-Derived Oligosaccharides. J Biochem (2007) 141:653–60. doi: 10.1093/jb/mvm067 17317686

[18] Kotani T Masutani R Suzuka T Oda K Makino S Ii M. Anti-Inflammatory and Anti-Fibrotic Effects of Intravenous Adipose-Derived Stem Cell Transplantation in a Mouse Model of Bleomycin-Induced Interstitial Pneumonia. Sci Rep (2017) 7:14608. doi: 10.1038/s41598-017-15022-3 29097816 PMC5668313

[19] Wellmann U Letz M Schneider A Amann K Winkler TH. An Ig Mu-Heavy Chain Transgene Inhibits Systemic Lupus Erythematosus Immunopathology in Autoimmune (NZB X NZW)F1 Mice. Int Immunol (2001) 13:1461–9. doi: 10.1093/intimm/13.12.1461 11717187

[20] Cunnane G Chan OT Cassafer G Brindis S Kaufman E Yen TS. Prevention of Renal Damage in Murine Lupus Nephritis by CTLA-4Ig and Cyclophosphamide. Arthritis Rheum (2004) 50:1539–48. doi: 10.1002/art.20147 15146424

[21] Bao L Haas M Kraus DM Hack BK Rakstang JK Holers VM. Administration of a Soluble Recombinant Complement C3 Inhibitor Protects Against Renal Disease in MRL/lpr Mice. J Am Soc Nephrol (2003) 14:670–9. doi: 10.1097/01.asn.0000051597.27127.a1 12595503

[22] Ling L Camilleri ET Helledie T Samsonraj RM Titmarsh DM Chua RJ. Effect of Heparin on the Biological Properties and Molecular Signature of Human Mesenchymal Stem Cells. Gene (2016) 576(1 Pt 2):292–303. doi: 10.1016/j.gene.2015.10.039 26484394 PMC5330685

[23] Forte G Minieri M Cossa P Antenucci D Sala M Gnocchi V. Hepatocyte Growth Factor Effects on Mesenchymal Stem Cells: Proliferation, Migration, and Differentiation. Stem Cells (2006) 24:23–33. doi: 10.1634/stemcells.2004-0176 16100005

[24] Liu L Chen JX Zhang XW Sun Q Yang L Liu A. Chemokine Receptor 7 Overexpression Promotes Mesenchymal Stem Cell Migration and Proliferation *via* Secreting Chemokine Ligand 12. Sci Rep (2018) 8:204. doi: 10.1038/s41598-017-18509-1 29317710 PMC5760632

[25] Tripathi V Kumar R Dinda AK Kaur J Luthra K. CXCL12-CXCR7 Signaling Activates ERK and Akt Pathways in Human Choriocarcinoma Cells. Cell Commun Adhes (2014) 21:221–8. doi: 10.3109/15419061.2013.876013 24450273

[26] Fenton K Fismen S Hedberg A Seredkina N Fenton C Mortensen ES. Anti-dsDNA Antibodies Promote Initiation, and Acquired Loss of Renal Dnase1 Promotes Progression of Lupus Nephritis in Autoimmune (NZBxNZW)F1 Mice. PloS One (2009) 4:e8474. doi: 10.1371/journal.pone.0008474 20041189 PMC2793523

[27] Bethunaickan R Sahu R Liu Z Tang YT Huang W Edegbe O. Anti-Tumor Necrosis Factor α Treatment of Interferon-α-Induced Murine Lupus Nephritis Reduces the Renal Macrophage Response But Does Not Alter Glomerular Immune Complex Formation. Arthritis Rheum (2012) 64:3399–408. doi: 10.1002/art.34553 PMC3443508 22674120

[28] Schena F Gambini C Gregorio A Mosconi M Reverberi D Gattorno M. Interferon-γ-Dependent Inhibition of B Cell Activation by Bone Marrow-Derived Mesenchymal Stem Cells in a Murine Model of Systemic Lupus Erythematosus. Arthritis Rheum (2010) 62:2776–86. doi: 10.1002/art.27560 20496367

[29] Thiel A Yavanian G Nastke MD Morales P Kouris NA Kimbrel EA. Human Embryonic Stem Cell-Derived Mesenchymal Cells Preserve Kidney Function and Extend Lifespan in NZB/W F1 Mouse Model of Lupus Nephritis. Sci Rep (2015) 5:17685. doi: 10.1038/srep17685 26628350 PMC4667213

[30] Anderson P Souza-Moreira L Morell M Caro M O'Valle F Gonzalez-Rey E. Adipose-Derived Mesenchymal Stromal Cells Induce Immunomodulatory Macrophages Which Protect From Experimental Colitis and Sepsis. Gut (2013) 62:1131–41. doi: 10.1136/gutjnl-2012-302152 22637701

[31] Singh RR. SLE: Translating Lessons From Model Systems to Human Disease. Trends Immunol (2005) 26:572–9. doi: 10.1016/j.it.2005.08.013 PMC2291517 16153890

[32] Jiang Z Sui T Wang B. Relationships Between MMP-2, MMP-9, TIMP-1 and TIMP-2 Levels and Their Pathogenesis in Patients With Lupus Nephritis. Rheumatol Int (2010) 30:1219–26. doi: 10.1007/s00296-009-1135-9 19779723

[33] Tveita AA Rekvig OP Zykova SN. Increased Glomerular Matrix Metalloproteinase Activity in Murine Lupus Nephritis. Kidney Int (2008) 74:1150–8. doi: 10.1038/ki.2008.308 18596727

[34] Uysal CA Tobita M Hyakusoku H Mizuno H. The Effect of Bone-Marrow-Derived Stem Cells and Adipose-Derived Stem Cells on Wound Contraction and Epithelization. Adv Wound Care (New Rochelle) (2014) 3:405–13. doi: 10.1089/wound.2014.0539 PMC4048969 24940554

[35] Giannopoulou M Dai C Tan X Wen X Michalopoulos GK Liu Y. Hepatocyte Growth Factor Exerts Its Anti-Inflammatory Action by Disrupting Nuclear factor-kappaB Signaling. Am J Pathol (2008) 173:30–41. doi: 10.2353/ajpath.2008.070583 18502824 PMC2438283

[36] Coudriet GM He J Trucco M Mars WM Piganelli JD. Hepatocyte Growth Factor Modulates Interleukin-6 Production in Bone Marrow Derived Macrophages: Implications for Inflammatory Mediated Diseases. PloS One (2010) 5:e15384. doi: 10.1371/journal.pone.0015384 21072211 PMC2970559

[37] Chen PM Liu KJ Hsu PJ Wei CF Bai CH Ho LJ. Induction of Immunomodulatory Monocytes by Human Mesenchymal Stem Cell-Derived Hepatocyte Growth Factor Through ERK1/2. J Leukoc Biol (2014) 96:295–303. doi: 10.1189/jlb.3A0513-242R 24714552

[38] Zhuang L Xia W Hou M. Co-culturing With Hypoxia Pre-Conditioned Mesenchymal Stem Cells as a New Strategy for the Prevention of Irradiation-Induced Fibroblast-to-Myofibroblast Transition. Oncol Rep (2019) 42:1781–92. doi: 10.3892/or.2019.7293 PMC6775806 31485596

[39] Wu MH Yokozeki H Takagawa S Yamamoto T Satoh T Kaneda Y. Hepatocyte Growth Factor Both Prevents and Ameliorates the Symptoms of Dermal Sclerosis in a Mouse Model of Scleroderma. Gene Ther (2004) 11:170–80. doi: 10.1038/sj.gt.3302165 14712301

[40] Kollet O Shivtiel S Chen YQ Suriawinata J Thung SN Dabeva MD. HGF, SDF-1, and MMP-9 Are Involved in Stress-Induced Human CD34+ Stem Cell Recruitment to the Liver. J Clin Invest (2003) 112:160–9. doi: 10.1172/JCI17902 PMC164291 12865405

[41] Liu H Liu S Li Y Wang X Xue W Ge G. The Role of SDF-1-CXCR4/CXCR7 Axis in the Therapeutic Effects of Hypoxia-Preconditioned Mesenchymal Stem Cells for Renal Ischemia/Reperfusion Injury. PloS One (2012) 7:e34608. doi: 10.1371/journal.pone.0034608 22511954 PMC3325280

[42] Sun L Wang D Liang J Zhang H Feng X Wang H. Umbilical Cord Mesenchymal Stem Cell Transplantation in Severe and Refractory Systemic Lupus Erythematosus. Arthritis Rheum (2010) 62:2467–75. doi: 10.1002/art.27548 20506343

[43] Wang D Zhang H Liang J Li X Feng X Wang H. Allogeneic Mesenchymal Stem Cell Transplantation in Severe and Refractory Systemic Lupus Erythematosus: 4 Years of Experience. Cell Transplant (2013) 22:2267–77. doi: 10.3727/096368911X582769c PMC11849135 24388428

[44] Gu F Wang D Zhang H Feng X Gilkeson GS Shi S. Allogeneic Mesenchymal Stem Cell Transplantation for Lupus Nephritis Patients Refractory to Conventional Therapy. Clin Rheumatol (2014) 33:1611–9. doi: 10.1007/s10067-014-2754-4 25119864

[45] Neubert K Meister S Moser K Weisel F Maseda D Amann K. The Proteasome Inhibitor Bortezomib Depletes Plasma Cells and Protects Mice With Lupus-Like Disease From Nephritis. Nat Med (2008) 14:748–55. doi: 10.1038/nm1763 18542049

[46] Choi EW Shin IS Park SY Park JH Kim JS Yoon EJ. Reversal of Serologic, Immunologic, and Histologic Dysfunction in Mice With Systemic Lupus Erythematosus by Long-Term Serial Adipose Tissue-Derived Mesenchymal Stem Cell Transplantation. Arthritis Rheum (2012) 64:243–53. doi: 10.1002/art.33313 21904997

[47] Wysoczynski M Khan A Bolli R. New Paradigms in Cell Therapy: Repeated Dosing, Intravenous Delivery, Immunomodulatory Actions, and New Cell Types. Circ Res (2018) 123:138–58. doi: 10.1161/CIRCRESAHA.118.313251 PMC6050028 29976684

