# Supplementary figures and images for: Low-Molecular-Weight Heparin Enhanced Therapeutic Effects of Human Adipose-Derived Stem Cell Administration in a Mouse Model of Lupus Nephritis
Source: Front Immunol. 2022 Jan 13;12:792739. doi: 10.3389/fimmu.2021.792739 (PMC8792143; doi:10.3389/fimmu.2021.792739)

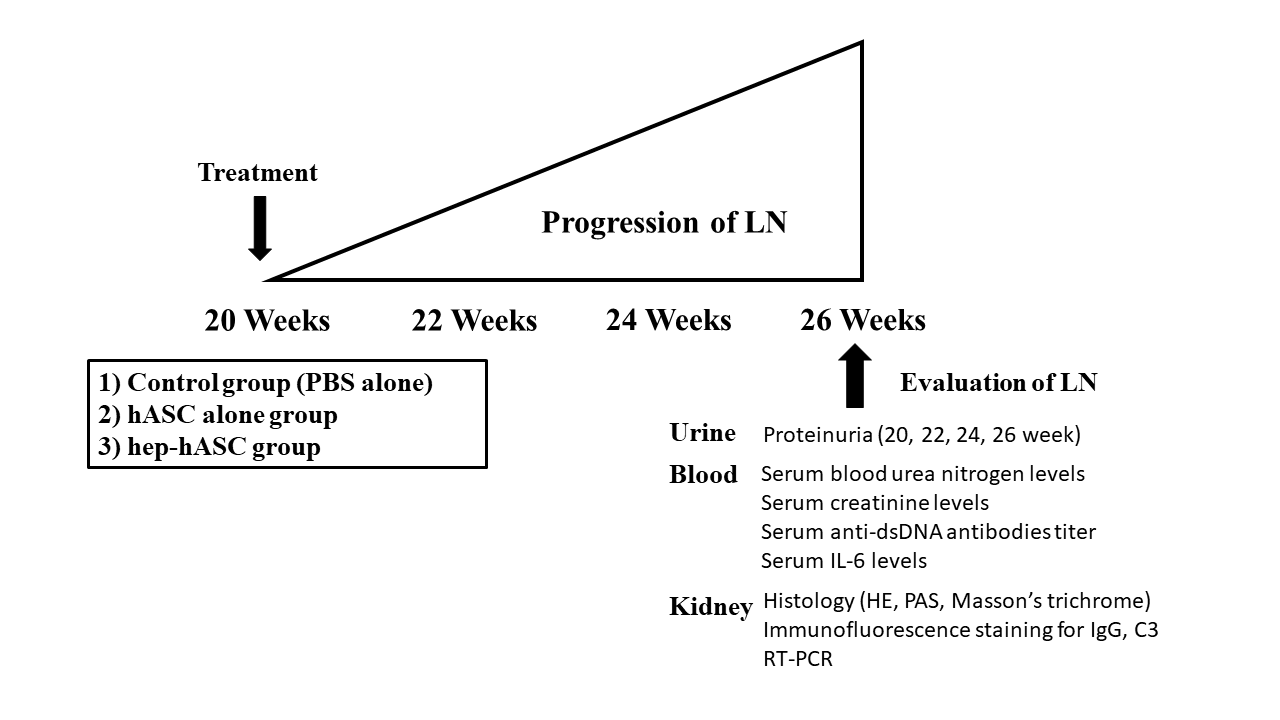

Supplement: Supplementary Figure 1 — Experimental protocol. 20-week-old female NZB/W F1 mice were randomized to receive saline, hASCs alone and hep-hASCs and they were followed for a further 6 weeks. Mice were euthanized and blood, urine and kidneys were collected for clinical, serological, and histological analysis. ASCs, adipose-derived mesenchymal stem cells. [file Image_1.tif]

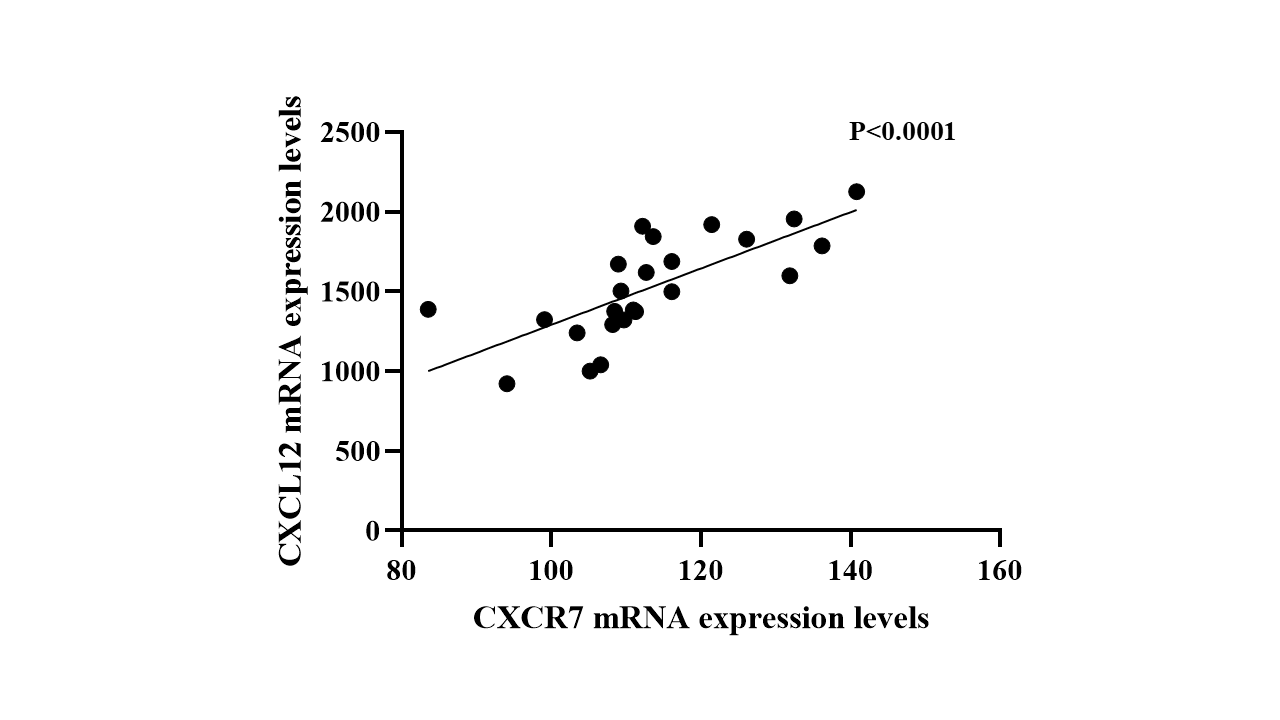

Supplement: Supplementary Figure 2 — Correlation of gene expressions in hASCs activated by LMWH. Correlation of CXCR7 mRNA expression levels with CXCL12 mRNA expression levels in hASCs. (N=24). [file Image_2.tif]

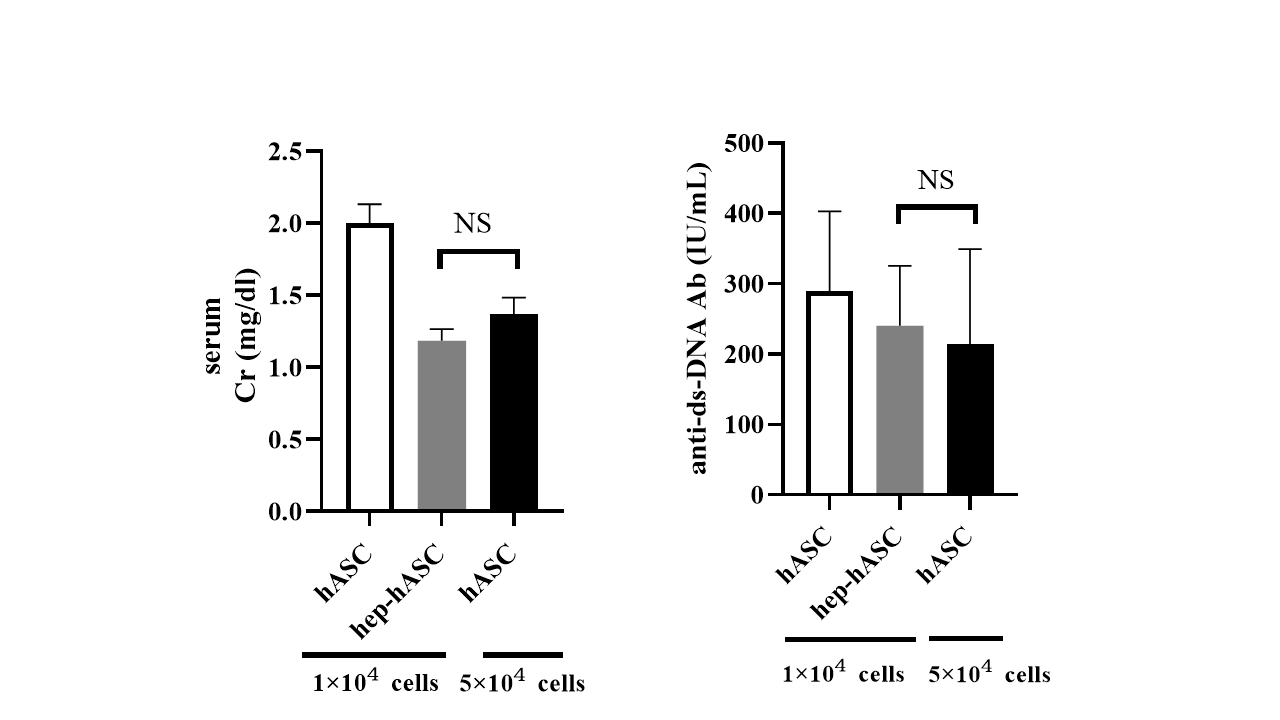

Supplement: Supplementary Figure 3 — Evaluation of therapeutic effects of ASC on lupus nephritis Comparison of kidney function between NZB/W F1 treated with 1×10^4^ hASC (N=12), 5×10^4^ hASC, (N=5) and 1×10^4^ hep-hASC (N=12). (A) Serum creatinine levels. (B) Serum anti-dsDNA antibody levels. [file Image_3.tif]
