# Supplementary material for: Low-Molecular-Weight Heparin Enhanced Therapeutic Effects of Human Adipose-Derived Stem Cell Administration in a Mouse Model of Lupus Nephritis
Source: Front Immunol. 2022 Jan 13;12:792739. doi: 10.3389/fimmu.2021.792739 (PMC8792143; doi:10.3389/fimmu.2021.792739)
Supplement: Supplementary file 4 [file Table_1.docx]

**Supplementary Table1. Primers used in RT-PCR analysis in vitro.**

| **Gene** | **Forward** | **Reverse** |
| --- | --- | --- |
| **CXCR7** | 5’-AGAAGATGGTACGCCGTGTCG-3’ | 5’-TCTTCCGGCTGCTGTGCTTCTC-3’ |
| **CXCR4** | 5’-TCATCAAGCAAGGGTGTGAG-3’ | 5’-GGCTCCAAGGAAAGCATAGA-3’ |
| **HGF** | 5’-CCTCTATGAAAACAAAGACTAC-3’ | 5’-CTGTGTTCGTGTGGTATC-3’ |
| **CXCL12** | 5’-GCTTTGAGTGACTGGGTT-3’ | 5’-GTGGCAAGATGATGGTTT-3’ |
| **PI3K** | 5’-ACCAGCACTGCCTCCTAAAC-3’ | 5’-TCTTCATCATCTTCCACCAGTG-3’ |
| **AKT** | 5’-ACTCTTTCCAGACCCACGACC-3’ | 5’-CAAAGAAGCGATGCTGCATG-3’ |
| **GAPDH** | 5’-TATGACAACAGCCTCAAG-3’ | 5’-ATGAGTCCTTCCACGATA-3’ |
