# Supplementary material for: Low-Molecular-Weight Heparin Enhanced Therapeutic Effects of Human Adipose-Derived Stem Cell Administration in a Mouse Model of Lupus Nephritis
Source: Front Immunol. 2022 Jan 13;12:792739. doi: 10.3389/fimmu.2021.792739 (PMC8792143; doi:10.3389/fimmu.2021.792739)
Supplement: Supplementary file 5 [file Table_2.docx]

**Supplementary Table2. Primers used in RT-PCR analysis in vivo.**

| **Gene** | **Forward** | **Reverse** |
| --- | --- | --- |
| **TIMP-2** | 5'-CTCGGAGCGCAATAAAACGG-3’ | 5'-CCTCTTGATGGGGTTGCCAT-3’ |
| **MMP-2** | 5’-AACCTCTTTGTGCTGAAA-3’ | 5’-GATGGTGTTCTGGTCAAG-3’ |
| **TNF-α** | 5’-ACCTTGTTGCCTCCTCTT-3’ | 5’-GTTCAGTGATGTAGCGACAG-3’ |
| **IL-2** | 5’-TGTGTAGGTAGACTCATTA-3’ | 5’-TTAGAGGAGAGCTTTATTTC-3’ |
| **IL-4** | 5’-TTAGCATCTCTTGATAAACT-3’ | 5’-ATATGGCTCCTGGTACAT-3’ |
| **IL-10** | 5’-GTGGAGCAGGTGAAGAGTGA-3’ | 5’-TTCATGGCCTTGTAGACACCT-3’ |
| **GAPDH** | 5’-ACAATGAATACGGCTACAG-3’ | 5’-GGTCCAGGGTTTCTTACT -3’ |
